# Global burden of chronic kidney disease due to hypertension attributable to dietary risks in adults aged 25 years and older: findings from the global burden of disease study 2021

**DOI:** 10.3389/fnut.2025.1593057

**Published:** 2025-07-24

**Authors:** Lianjie Huang, Yujie Qiu, Zheng Long, Caicui Ding, Weiyan Gong, Ailing Liu

**Affiliations:** ^1^National Institute for Nutrition and Health, Chinese Center for Disease Control and Prevention, Beijing, China; ^2^Department of Medical Affairs, Xuanwu Hospital Capital Medical University, Beijing, China

**Keywords:** global burden of disease study, chronic kidney disease, hypertension, dietary risks, mortality, disability-adjusted life years

## Abstract

**Background:**

Suboptimal diet remains a major threat to chronic kidney diseases (CKD) and hypertension, however, the burden of diet-attributable CKD due to hypertension (HCKD) has been poorly quantified and reported. This study aimed to provide a comprehensive and up-to-date view of global temporal and geographical trends in diet-attributable HCKD burden.

**Methods:**

Data on diet-attributable HCKD burden were extracted from the Global Burden of Disease Study (GBD) 2021. Number and age-standardized rates (ASR) of mortality and disability-adjusted life years (DALYs) with their average annual percentage change (AAPC) were used to describe the diet-attributable HCKD burden. Decomposition analysis was employed to assess the contributions of aging, population, and epidemiological changes to HCKD burden.

**Results:**

Globally, the ASR of mortality (ASMR) and DALYs (ASDR) of diet-attributable HCKD among adults aged ≥25 years increased from 3.7 (95% uncertainty interval [UI]: 2.0–5.6) and 91.7 (50.1–140.1) per 100,000 population in 1990 to 4.3 (2.3–6.7) and 101.2 (56.0–154.5) per 100,000 population in 2021, respectively. Both mortality and DALYs of diet-attributable HCKD burden in 2021 were higher among males than females. Low fruit and low vegetable intake contributed the most to the diet-attributable HCKD burden. The highest ASMR and ASDR of diet-attributable HCKD were observed in Central Sub-Saharan Africa, whereas the lowest were observed in Eastern Europe. The older adult population had higher mortality and DALYs than other age groups. Decomposition analysis showed that population growth mainly contributed to HCKD burden, particularly in low SDI regions.

**Conclusion:**

The diet-attributable HCKD burden is increasing at an alarming rate globally, especially in low SDI countries and older adults. This study emphasizes the urgent need for rigorous public health interventions to change unhealthy dietary behaviors and decrease diet-attributable HCKD burden worldwide.

## 1 Introduction

Chronic kidney disease (CKD) is a progressive disease characterized by a gradual loss of kidney function over time (1) and has become a global public health concern, with approximately 700 million individuals with all-stage CKD in 2017 worldwide (2). In 2040, CKD will become the fifth leading cause of mortality with 3.1 million deaths globally and one of the fastest increasing trends in total major causes of death (3). Among many established causes for CKD, hypertension was considered one of the leading drivers, which had increased significantly over the past 30 years. Across the globe, CKD due to hypertension (HCKD) collectively accounts for 23.26% of the increased burden of overall CKD (4). Simultaneously, the disease burden of hypertension is showing an uncontrollable upward trend (5). The boosted burden of CKD might be largely caused by the increased global epidemic of hypertension. The research on HCKD burden has not been reported in depth in the past decades. It illustrates that hypertension is the leading driver of CKD, and little progress has been made in the treatment and control of the HCKD burden worldwide.

Dietary risk was the fifth leading risk factor to global DALYs in 2021 and gained in prominence for those aged 50–69 years (6). Increased sugar-sweetened beverages consumption and dietary patterns characterized by excess intake of deep-fried foods, processed, and organ meats were positively associated with a higher risk of CKD (7). Conversely, sufficient consumption of fruits, vegetables, whole grains, and low-fat dairy would reduce the urinary albumin and creatinine ratio according to an American cohort study (8). The higher intake of plant-based food (including whole grains, fruits, vegetables, etc) had the potential role of mitigating unfavorable health outcomes (9).

As a chronic disease as severe as cancer and cardiovascular disease, the mortality risk and burden of CKD are rising sharply in middle- and low-income countries and regions because of its expensive cost of dialysis and kidney transplantation, and limited capacity of delivering care for kidney failure (10). The CKD costs exceeding 114 billion dollars in the USA and 1.45 billion pounds in the UK annually, while most people in low- or middle-income countries cannot afford kidney therapy (11). Socioeconomic status is a major reason contributing to the inequality of CKD burden. This may be attributed to the fact that higher educational attainment provides greater capabilities of decision-making and enables them to select a healthier lifestyle. Hence, CKD burden is disproportionately higher in less well-developed economies with poor health systems.

The present study aims to offer a detailed depiction of the HCKD landscape globally attributable to seven dietary risk factors, promote a deeper perception of the effects of dietary risk in terms of mortality and disability-adjusted life years (DALYs), and elucidate the trends from 1990 to 2021.

## 2 Materials and methods

### 2.1 Data sources and definitions

The Global Burden of Disease Study 2021 (GBD 2021) is a collaborative integrated surveillance system that provides up-to-date descriptive epidemiological data of 369 diseases and injuries, using comparative risk assessment, with 87 risk factors for 204 countries and territories from 1990 to 2021 (12). This study was hosted by the Institute for Health Metrics and Evaluation and could be available at https://vizhub.healthdata.org/gbd-results/.

### 2.2 Estimation of disease and diet-attributable burden

In GBD database, CKD is categorized into total CKD (code B.8.2 “chronic kidney disease”) and five specific subtypes (including B.8.2.1 “chronic kidney disease due to diabetes mellitus type 1,” B.8.2.2 “chronic kidney disease due to diabetes mellitus type 2,” B.8.2.3 “chronic kidney disease due to hypertension,” B.8.2.4 “chronic kidney disease due to glomerulonephritis,” B.8.2.5 “chronic kidney disease due to other and unspecified causes”) according to the GBD cause list following the International Classification of Diseases and Injuries (ICD-10) codes (2). In our study, we selected “chronic kidney disease due to hypertension” as the major cause. Data on disease burden and exposure of dietary risk factors in GBD 2021 were modelled and quantified using the Comparative Risk Assessment Framework and DisMod-MR 2.1, which are Bayesian statistical models developed for GBD analysis (13).

### 2.3 Selection of dietary risk factors

The GBD comparative risk assessment framework was used to compute the fraction of CKD-specific burden attributable to dietary risk factors. The estimation of mortality and DALYs attributable to total dietary risk and six specific dietary risk factors relevant to the outcomes of HCKD among adults (≥ 25 years) in global were acquired in this study, including diet low in fruit (low fruit), diet low in vegetable (low vegetable), diet low in whole grain (low whole grain), diet high in processed meat (high processed meat), diet high in sugar-sweetened beverages (high sugar beverages) and diet high in sodium (high sodium). In our current analysis, we have focused on dietary risk factors independently, without explicitly accounting for the potential interdependence of risk factors such as obesity or hypertension.

### 2.4 Statistical analysis

To measure the diet-attributable HCKD burden across different regions, the Socio-Demographic Index (SDI) was used to estimate the development status of geographical location based on a composite of country-level income per capita, average years of schooling, and total fertility rate. The SDI ranges from 0 to 1, with higher values indicating higher socioeconomic levels. The dataset cover various demographic groups in 204 countries, 21 global regions and 5 Socio-Demographic Index regions, including High SDI (>0.81), High-middle SDI (0.70–0.81), Middle SDI (0.61–0.69), Low-middle SDI (0.46–0.60), Low SDI (<0.46) (14). The Joinpoint regression model was used to calculate the temporal trend changes in diet-attributable CKD burden performing with the Joinpoint Regression Program software (version 5.1.0.0, Statistical Research and Applications Branch, National Cancer Institute) based on the Monte Carlo permutation method. AAPC was used to represent the average annual change trend of rates over the entire period, which was transformed from the weighted average of the slope coefficients from 1990 to 2021 (15). The AAPC value indicates three statuses of percentage annual change, including ascending, descending, and no change. The corresponding rate was considered as an ascending trend (or descending) if the AAPC value and its 95% confidence intervals (95% CI) were both >0 (or both <0), while *p* value > 0.05 was considered as no change. Additionally, the decomposition method was utilized to break down the changes in diet-attributable burden into contributions from aging, population, and epidemiological changes from 1990 to 2021.

In this study, a range of variables were employed to precisely examine the diet-attributable HCKD burden, including the number and ASR with its 95% uncertainty intervals (95% UI). Furthermore, we analyzed 15 age groups, ranging from 25–29 to ≥95 years in 5-year intervals. The ASMR (per 100,000 population) and ASDR (per 100,000 population) with their 95% UIs were calculated by the following algorithm:


$$\mathrm{ASR}=\frac{\sum_{i=1\;}^{n}{\mathrm{age}}_{i}*{\text{Standard}}_{i}}{\sum_{i=1\;}^{n}{\text{Standard}}_{i}}$$

where age represents the age-specific rate with its 95% UI, standard represents the population weight in the same age group of the reference standard population, i represents the age group number in this study (range 1 to 15) (15).

All statistical analysis and data visualizations of this study were carried out in R software (version 4.2.3) using the package “ggplot2” and “dplyr.” The two-tailed test with *p*-value < 0.05 was considered statistically significant.

## 3 Results

### 3.1 Global trends in HCKD burden attributable to dietary risks from 1990 to 2021

In 2021, the mortality and DALYs numbers of diet-attributable HCKD were 193.9 thousand (95% UI: 104.3–301.3) and 4.7 million (95% UI: 2.6–7.2), with an increase of 176.0 and 136.8% from 1990, respectively. The ASMR and ASDR of diet-attributable HCKD were 4.3 (95% UI: 2.3–6.7) and 101.2 (95% UI: 56.0–154.5) per 100,000 population, with an AAPC of 0.5% (95% CI: 0.4–0.6%) and 0.3% (95% CI: 0.2–0.4%) from 1990 to 2021, respectively. Significant geographic disparities were observed across different SDI regions in 2021, with the biggest mortality and DALYs number recorded in Middle SDI regions (71.5 thousand [95% UI: 36.6–113.8] and 1.8 million [95% UI: 1.0–2.8], respectively), and the highest ASMR and ASDR were recorded in Low SDI regions (8.4 [95% UI: 4.5–13.0] and 180.6 [95% UI: 98.9–277.4], respectively).

**Figure 1:**
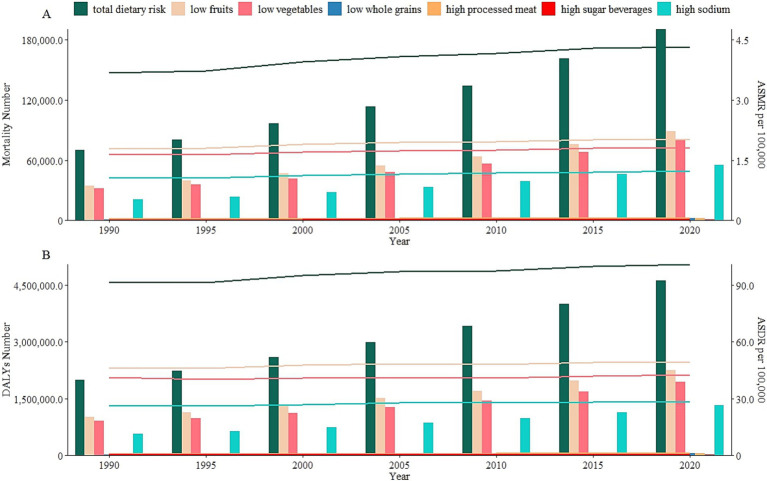
Number and age-standardized rates of diet-attributable HCKD burden in seven dietary risks from 1990 to 2021. **(A)**. Mortality. **(B)**. DALYs.

By specific dietary risk, the mortality and DALYs numbers of diet-attributable HCKD have increased gradually over time, reaching their highest point in 2021. However, the ASMR and ASDR of diet-attributable HCKD have remained stable from 1990 to 2021. The low fruit intake contributed to the highest burden in both mortality and DALYs. From 1990 to 2021, the mortality numbers attributable to low fruit intake increased significantly by approximately 164%, rising from 34.3 thousand (95% UI: 16.7–56.3) in 1990 to 90.5 thousand (95% UI: 46.1–142.2) in 2021. Similarly, DALYs caused by low fruit consumption increased by 127%, from 1.0 million (95% UI: 0.5–1.7) in 1990 to 2.3 million (95% UI: 1.2–3.6) in 2021. Meanwhile, high sugar beverages had the biggest increasing trend of ASMR and ASDR from 1990 to 2021 (AAPC: 2.4 and 2.8%, respectively) (Table 1, Figure 1, Supplementary Tables S1–S3, Supplementary Figure S1).

**Table 1:** Global number, age-standardized rates and average annual percentage changes for diet-attributable HCKD burden from 1990 to 2021.

Dietary risks	1990		2021		1990–2021
	Number No. × 10^3^ (95%UI)	ASR per 10^5^ (95%UI)	Number No. × 10^3^ (95%UI)	ASR per 10^5^ (95%UI)	AAPC (95%CI)
Mortality					
Total dietary risk	70.3 (37.2, 110.9)	3.7 (2.0, 5.6)	193.9 (104.3, 301.3)	4.3 (2.3, 6.7)	0.5 (0.4, 0.6)^+^
Low fruit	34.3 (16.7, 56.4)	1.8 (0.9, 2.9)	90.5 (46.1, 142.2)	2.0 (1.0, 3.2)	0.4 (0.3, 0.5)^**+**^
Low vegetable	31.7 (15.2, 53.7)	1.7 (0.8, 2.8)	81.0 (40.6, 132.4)	1.8 (0.9, 2.9)	0.3 (0.2, 0.4)^**+**^
Low whole grain	0.5 (0, 1.7)	0 (0, 0.1)	2.3 (0.3, 6.2)	0.1 (0, 0.2)	1.4 (1.2, 1.5)^**+**^
High processed meat	0.5 (0, 1.6)	0 (0, 0.1)	2.3 (0.2, 6.3)	0.1 (0, 0.2)	1.8 (1.6, 2.0)^**+**^
High sugar beverages	0.1 (0, 0.4)	0 (0, 0.1)	0.8 (0.1, 2.0)	0 (0, 0.1)	2.4 (2.2, 2.7)^**+**^
high sodium	20.7 (3.2, 54.6)	1.1 (0.2, 2.8)	56.3 (6.3, 159.6)	1.2 (0.1, 3.5)	0.5 (0.4, 0.7)^**+**^
DALYs					
Total dietary risk	1996.3 (1081.1, 3070.4)	91.7 (50.1, 140.1)	4728.1 (2616.8, 7223.5)	101.2 (56.0, 154.5)	0.3 (0.2, 0.4)^+^
Low fruit	1013.3 (508.2, 1650.3)	46.0 (23.3, 74.3)	2306.1 (1198.7, 3603.8)	49.4 (25.7, 77.3)	0.2 (0.1, 0.3)^**+**^
Low vegetable	903.8 (436.9, 1525.6)	41.3 (20.2, 69.3)	1987.6 (1004.4, 3265.2)	42.6 (21.5, 70.1)	0.1 (0, 0.2)^**+**^
Low whole grain	11.4 (0.7, 37.9)	0.6 (0, 2.0)	44.9 (4.0, 128.9)	1.0 (0.1, 2.8)	1.6 (1.4, 1.7) ^**+**^
High processed meat	10.0 (0.5, 37.3)	0.6 (0, 1.9)	43.7 (3.3, 132.2)	1.0 (0.1, 2.9)	1.8 (1.7, 2.0)^**+**^
High sugar beverages	3.1 (0.2, 10.3)	0.2 (0, 0.5)	17.0 (1.8, 47.8)	0.4 (0, 1.0)	2.8 (2.6, 2.9)^**+**^
High sodium	569.5 (97.7, 1454.2)	26.3 (4.4, 67.6)	1355.7 (178.6, 3672.6)	28.7 (3.7, 78.1)	0.3 (0.2, 0.4)^**+**^

^+^ means *P*-value < 0.001.

### 3.2 Diet-attributable HCKD burden among different countries and territories

Among 204 countries and territories of diet-attributable HCKD burden in 2021, China, USA and India were the top three countries with the highest mortality number, with each exceeding 21,000. India and China were the top two countries with the highest DALYs number globally, with each exceeding 590,000. The highest absolute disease burden in these countries is primarily driven by their large population sizes. After standardizing for age structure across countries, Mauritius had the biggest ASMR and ASDR caused by diet-attributable HCKD in 2021 (29.1 [95% UI: 13.8–47.0] and 660.4 [95% UI: 311.0–1,078.1], respectively). Regarding the increasing trend of ASMR, Ukraine had the biggest AAPC with HCKD (9.47 [95% CI: 7.0–12.0]). Meanwhile, while United Arab Emirates had the biggest AAPC of ASDR caused by HCKD (3.8 [95% CI: 1.8–5.8]) (Supplementary Tables S6).

**Figure 2:**
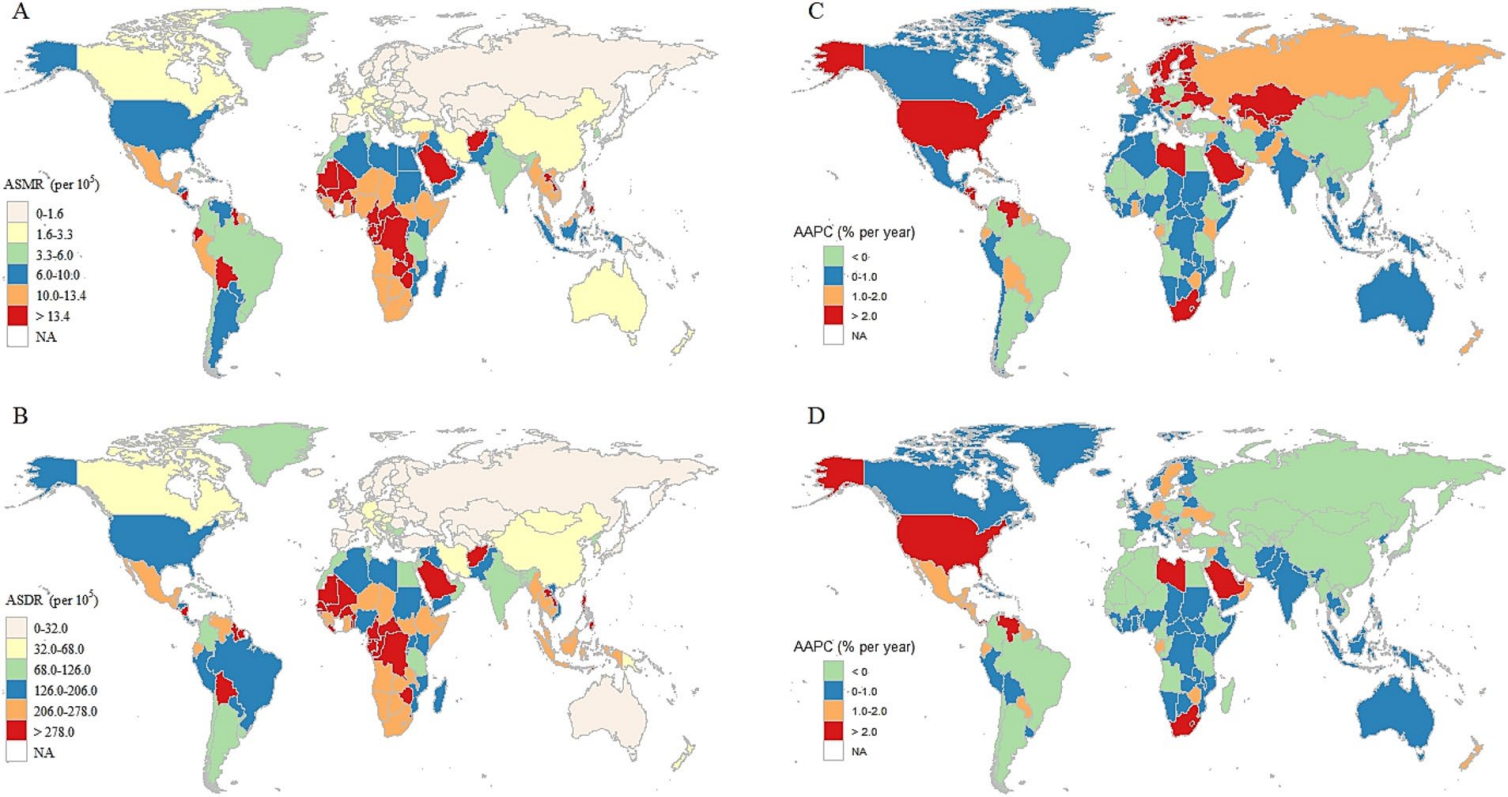
The global burden map of diet-attributable HCKD across 204 countries and territories in 2021. **(A)**. ASMR. **(B)**. ASDR. **(C)**. AAPC of ASMR. **(D)**. AAPC of ASDR.

Geographically, considerable disparities exist in the trends of diet-attributable HCKD burden across different regions. The highest mortality and DALYs of diet-attributable HCKD were observed in Southern Asia (33.7 thousand [95% UI: 16.5–54.4]) and South Asia (0.9 million [95% UI: 0.5–1.4]). Meanwhile, the highest ASMR and ASDR of diet-attributable HCKD were simultaneously observed in the Central Sub-Saharan Africa (14.0 [95% UI: 6.7–23.7] and 294.3 [95% UI: 144.7–495.1], respectively). Notably, the highest AAPC of ASMR and ASDR were observed in High-income North America (3.0% [95% CI: 2.7–3.2%] for ASMR and 2.7% [95% CI: 2.5–3.0%] for ASDR), followed by Central Asia (2.3% [95% CI: 1.3–3.3%] for ASMR) and Southern Sub-Saharan Africa (1.9% [95% CI: 1.6–2.2%] for ASDR). Conversely, the largest annual decrease trend was observed in East Asia (−1.6% [95% CI: −1.8% to −1.4%] for ASMR and −1.7% [95% CI: −1.8% to −1.6%] for ASDR) (Table 2) (Figure 2).

**Table 2:** Number and percentage change, age-standardized rates and average annual percentage changes for diet-attributable HCKD burden from 1990 to 2021.

Regions	1990		2021		1990–2021
	Number No. × 10^3^ (95%UI)	ASR per 10^5^ (95%UI)	Number No. × 10^3^ (95%UI)	ASR per 10^5^ (95%UI)	AAPC (95%CI)
Mortality					
Global	70.3 (37.2, 110.9)	3.7 (2.0, 5.7)	193.9 (104.3, 301.3)	4.3 (2.3, 6.7)	0.5 (0.4, 0.6)^+^
East Asia	14.0 (6.1, 25.0)	3.8 (1.7, 6.7)	25.3 (9.5, 48.8)	2.3 (0.9, 4.5)	−1.6 (−1.8, −1.4)^+^
Southeast Asia	12.0 (5.9, 19.2)	9.8 (4.9, 15.6)	33.7 (16.5, 54.4)	10.7 (5.3, 17.2)	0.3 (0.2, 0.4)^+^
Oceania	0 (0, 0)	1.7 (0.7, 3.4)	0.1 (0, 0.1)	2.1 (0.9, 4.0)	0.6 (0.5, 0.8)^+^
Central Asia	0.1 (0, 0.1)	0.2 (0.1, 0.4)	0.2 (0.1, 0.3)	0.4 (0.2, 0.8)	2.3 (1.3, 3.3)^+^
Central Europe	1.4 (0.8, 2.2)	1.9 (1.0, 3.0)	2.2 (1.2, 3.6)	1.7 (1.0, 2.8)	−0.2 (−0.8, 0.3)
Eastern Europe	0.5 (0.3, 0.8)	0.3 (0.2, 0.6)	1.1 (0.6, 1.8)	0.6 (0.3, 0.9)	1.6 (1.0, 2.3)^+^
High-income Asia Pacific	1.9 (1.1, 3.0)	2.0 (1.1, 3.2)	5.7 (2.8, 9.8)	1.5 (0.8, 2.7)	−0.8 (−1.3, −0.4)^+^
Australasia	0.2 (0.1, 0.2)	1.5 (0.9, 2.1)	0.7 (0.4, 1.1)	2.0 (1.0, 3.2)	0.9 (0, 1.8)
Western Europe	3.8 (2.1, 6.1)	1.2 (0.6, 1.9)	12.1 (6.8, 18.8)	1.7 (1.0, 2.7)	1.3 (0.9, 1.6)^+^
Southern Latin America	1.6 (0.8, 2.7)	7.0 (3.4, 11.5)	2.9 (1.4, 4.8)	5.9 (2.8, 9.6)	−0.6 (−1.1, −0.1)^*^
High-income North America	4.9 (2.6, 7.4)	2.5 (1.3, 3.7)	23.5 (12.4, 35)	6.1 (3.2, 9.0)	3.0 (2.7, 3.2)^+^
Caribbean	0.7 (0.4, 1.2)	5.6 (3.0, 8.9)	2.0 (1.1, 3.1)	6.6 (3.6, 10.4)	0.6 (0.3, 0.9)^+^
Andean Latin America	1.0 (0.5, 1.7)	10.0 (4.6, 16.6)	3.9 (1.8, 6.4)	12.5 (6.0, 20.5)	0.8 (0.4, 1.2)^+^
Central Latin America	2.6 (1.4, 4.1)	6.8 (3.7, 10.7)	12.1 (6.5, 19.3)	9.1 (4.9, 14.5)	0.9 (0.2, 1.6)^*^
Tropical Latin America	2.6 (1.5, 4.0)	6.2 (3.4, 9.4)	7.9 (4.3, 12.3)	5.8 (3.1, 9.1)	−0.1 (−0.4, 0.2)
North Africa and Middle East	4.0 (1.8, 7.1)	5.5 (2.5, 10.0)	10.5 (5.1, 17.4)	5.1 (2.5, 8.4)	−0.2 (−0.3, −0.1)^*^
South Asia	9.3 (4.9, 15.0)	3.4 (1.8, 5.4)	27.1 (14.6, 43.7)	3.8 (2.1, 6.1)	0.4 (0.1, 0.7)^*^
Central Sub-Saharan Africa	1.2 (0.6, 2.0)	13.1 (6.6, 21.4)	3.1 (1.5, 5.4)	14.0 (6.7, 23.7)	0.2 (0.1, 0.3)^+^
Eastern Sub-Saharan Africa	3.5 (1.7, 5.7)	10.7 (5.4, 17.6)	6.6 (3.5, 10.6)	9.6 (5.1, 15.3)	−0.4 (−0.5, −0.2)^+^
Southern Sub-Saharan Africa	0.8 (0.4, 1.3)	6.2 (3.2, 9.9)	3.2 (1.7, 4.9)	12.2 (6.6, 18.5)	2.1 (1.9, 2.4)^+^
Western Sub-Saharan Africa	4.3 (2.1, 6.9)	11.2 (5.5, 17.7)	10.3 (5.1, 16.2)	12.3 (6.2, 19.2)	0.3 (0.2, 0.4)^+^
DALYs					
Global	1996.3 (1081.1, 3070.4)	91.7 (50.1, 140.1)	4728.1 (2616.8, 7223.5)	101.2 (56.0, 154.5)	0.3 (0.2, 0.4)^+^
East Asia	416.4 (186.7, 731.2)	90.7 (41.2, 156.7)	623.8 (246.0, 1169.3)	53.5 (21.1, 100.3)	−1.7 (−1.8, −1.6)^+^
Southeast Asia	348.4 (170.9, 559.5)	237.0 (117.8, 375.8)	879.3 (430.4, 1425.6)	242.9 (119.6, 391.1)	0.1 (0, 0.2)^*^
Oceania	0.8 (0.3, 1.4)	48.4 (21.3, 86.5)	2.4 (1.1, 4.3)	57.8 (26.5, 103.2)	0.6 (0.4, 0.7)^+^
Central Asia	7.2 (4.5, 11.0)	27.0 (16.7, 40.8)	11.9 (7.1, 18.9)	26.3 (15.7, 41.7)	−0.1 (−0.2, 0.1)
Central Europe	36.9 (22.7, 55.5)	47.7 (29.3, 71.6)	48.0 (28.9, 74.6)	39.8 (24.1, 61.7)	−0.6 (−0.9, −0.2)^*^
Eastern Europe	29.1 (18.9, 42.9)	20.3 (13.1, 29.9)	38.2 (24.4, 57)	21.0 (13.4, 31.4)	0.1 (−0.2, 0.5)
High-income Asia Pacific	44.3 (27.4, 66.6)	42.3 (26.0, 64.0)	90.4 (48.9, 148.3)	31.4 (17.1, 50.9)	−1.0 (−1.4, −0.6)^+^
Australasia	3.3 (2.2, 4.6)	26.8 (17.6, 37.7)	10.4 (5.7, 16.2)	32.4 (18.0, 50.6)	0.6 (−0.2, 1.3)
Western Europe	86.0 (53.6, 129.2)	27.9 (17.3, 41.9)	190.7 (116.7, 283.9)	32.9 (20.1, 49.1)	0.6 (0.3, 0.8)^+^
Southern Latin America	34.1 (16.7, 55.3)	138.1 (67.8, 224.1)	51.2 (25.2, 83.7)	105.7 (52.1, 172.9)	−0.9 (−1.3, −0.4)^+^
High-income North America	102.5 (56.9, 151.2)	53.1 (29.3, 78.6)	428.8 (232.1, 628.3)	122.4 (66.3, 179.3)	2.7 (2.5, 3.0)^+^
Caribbean	19.4 (10.6, 30.2)	134.2 (73.1, 208.2)	46.8 (26.2, 72.9)	159.7 (89.2, 248.7)	0.6 (0.4, 0.8)^+^
Andean Latin America	23.7 (10.8, 39.5)	208.8 (96.0, 346.5)	79.7 (38.4, 131.3)	246.3 (119.0, 405.1)	0.7 (0.4, 1.0)^+^
Central Latin America	67.8 (39.0, 103.8)	148.5 (85.5, 226.2)	295.1 (164.8, 464.4)	212.4 (118.9, 333.6)	1.2 (0.6, 1.8)^+^
Tropical Latin America	77.8 (45.3, 116.4)	149.4 (86.9, 222.5)	181.1 (103.9, 275.8)	129.0 (73.8, 196.2)	−0.4 (−0.7, −0.2)^+^
North Africa and Middle East	100.1 (46.6, 174.3)	111.6 (52.0, 196.2)	254.5 (123.7, 424.3)	102.6 (50.4, 169.7)	−0.3 (−0.4, −0.1)^+^
South Asia	338.7 (188.6, 524.1)	99.6 (56.8, 151.8)	886.5 (512.1, 1367.5)	106.1 (62.0, 162.3)	0.2 (0, 0.4)^*^
Central Sub-Saharan Africa	34.7 (17.5, 57.0)	284.3 (144.4, 461.9)	90.4 (44.3, 154.3)	294.3 (144.7, 495.1)	0.1 (0, 0.2)^+^
Eastern Sub-Saharan Africa	91.0 (46.6, 149.3)	226.3 (116.1, 369.0)	164.8 (90.5, 264.3)	184.3 (101.0, 293.2)	−0.6 (−0.8, −0.5)^+^
Southern Sub-Saharan Africa	23.2 (12.0, 36.3)	146.7 (76.4, 228.5)	84.4 (45.7, 128.8)	262.5 (142.6, 396.8)	1.9 (1.6, 2.2)^+^
Western Sub-Saharan Africa	111.0 (52.7, 179.4)	232.6 (112.2, 371.1)	269.7 (133.3, 430.3)	245.9 (123.7, 385.2)	0.2 (0.1, 0.3)^+^

^+^ means *P*-value < 0.001, ^*^ means *P*-value < 0.05.

### 3.3 Diet-attributable HCKD burden across age and gender in 2021

**Figure 3:**
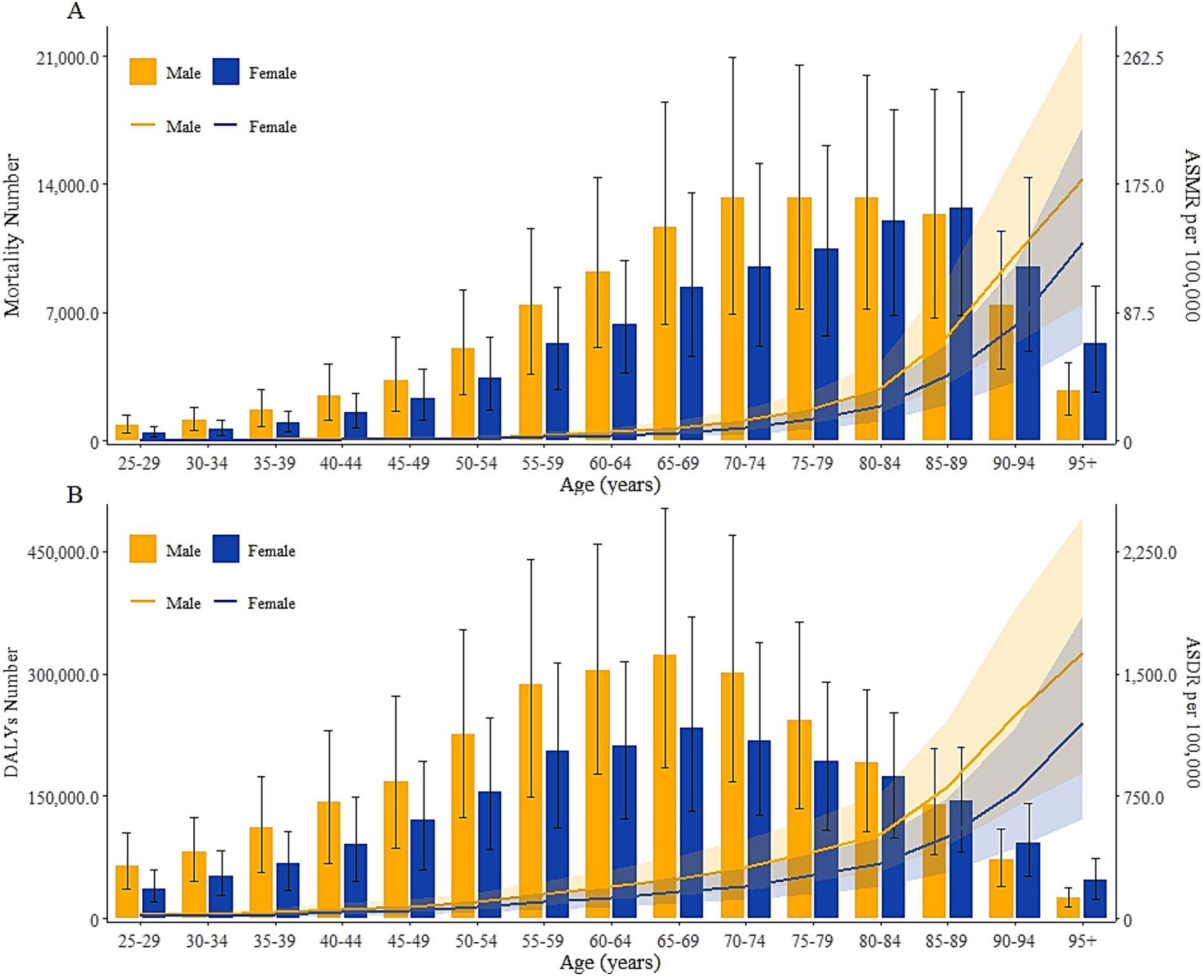
Number and age-standardized rates of diet-attributable HCKD burden across age and gender stratification in 2021. **(A)**. Mortality. **(B)**. DALYs.

Globally, the mortality and DALYs number of HCKD in 2021 were 105.0 thousand (95% UI: 55.4–164.8) and 2.7 million (95% UI: 1.5–4.1) for males,89.0 thousand (95% UI: 47.7–138.6) and 2.0 million (95% UI: 1.1–3.1) for females. In 2021, the mortality number of diet-attributable HCKD varied by age group, with a peak in males aged 70 to 74 and in females aged 85 to 89. Meanwhile, the peak of DALYs due to diet-attributable HCKD occurred in males and females aged 65 to 69. The ASMR and ASDR of HCKD increased with aging in all age groups (Figure 3). In addition, the mortality and DALYs in males were consistently higher than in females across all age groups below 80 years, while the burden in females was higher than in males after the age of 80 (Supplementary Tables S3, S4).

### 3.4 Decomposition analysis

**Figure 4:**
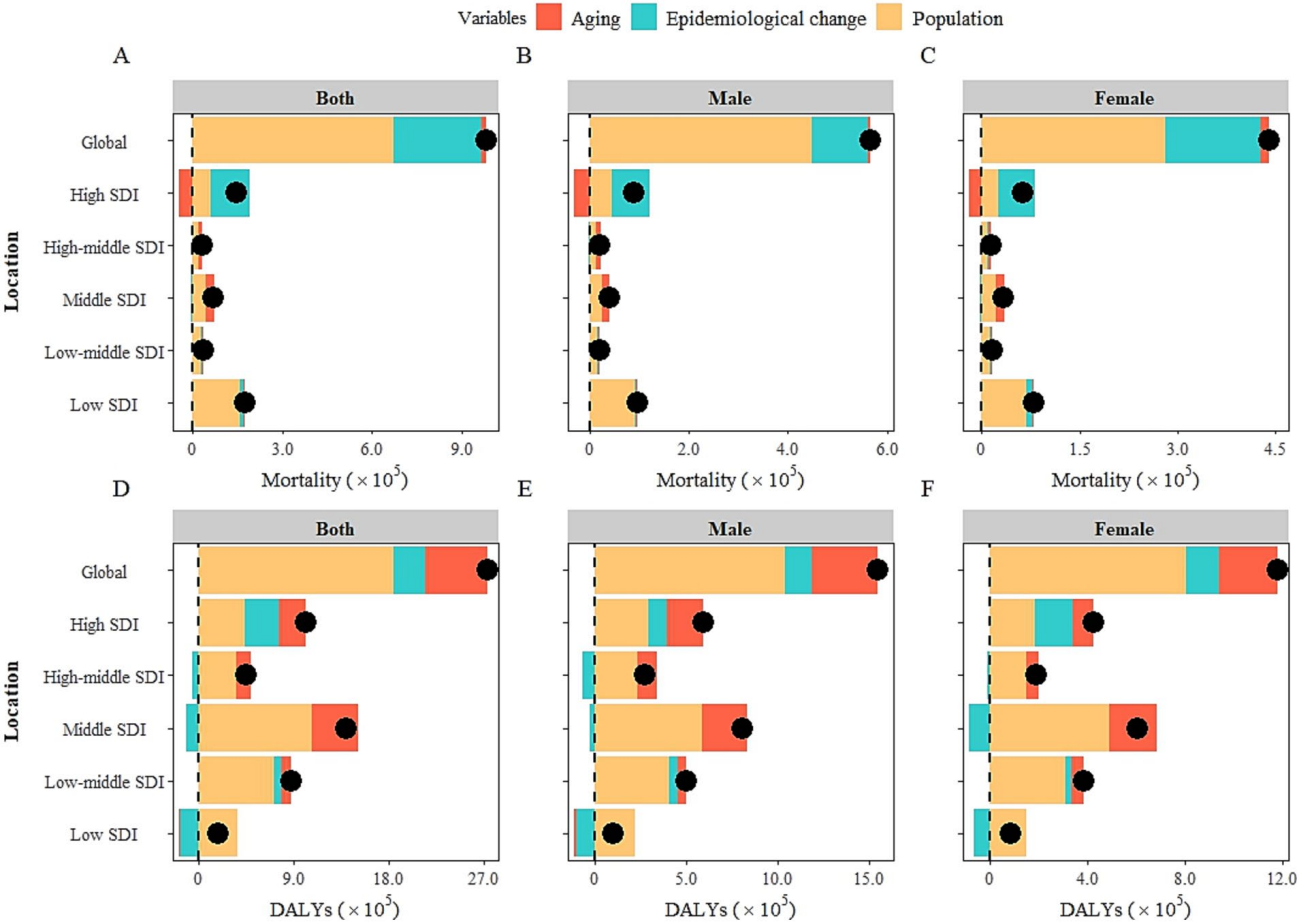
Changes in diet-attributable HCKD burden according to population-level determinants of population growth, aging and epidemiological change across SDI regions and gender from 1990 to 2021. **(A)**. Mortality of both sexes. **(B)**. Mortality of males. **(C)**. Mortality of females. **(D)**. DALYs of both sexes. **(E)**. DALYs of males. **(F)**. DALYs of females.

Decomposition analysis was utilized to evaluate the contributions of aging, population, and epidemiological changes on diet-attributable HCKD burden from 1990 to 2021 in this study. The results showed that aging, epidemiological change, and population growth contributed 1.8, 29.8, and 68.4% in mortality, 21.4, 10.9, and 67.7% in DALYs to the increase in diet-attributable HCKD burden globally, respectively. Aging had a positive impact on diet-attributable HCKD burden in regions with High-middle SDI, Middle SDI, and Low-middle SDI, indicating a marked effect of aging on HCKD burden in these regions. The highest contribution of epidemiological changes was observed in High SDI regions (88.4 and 31.4% for mortality and DALYs, respectively). Meanwhile, a reduction was observed in the Middle SDI regions (−5.9% and −8.1% for mortality and DALYs, respectively). This might suggest that countries in the Middle SDI regions have achieved relatively better control of the HCKD burden. Population growth exhibited an increasing trend of diet-attributable HCKD burden both globally and in different SDI regions, with proportions exceeding 40% (Figure 4, Supplementary Table S5).

## 4 Discussion

After a comprehensive analysis of diet-attributable HCKD burden globally from 1990 to 2021, our research provided straightforward insights that could be a foundation for future prevention to alleviate the burden of diet-attributable HCKD. This study illustrated a sharp increase in mortality and DALYs (both number and ASR) of diet-attributable HCKD over the past three decades, with a remarkable contribution from low fruit, low vegetable, and high sodium, while a severely increasing annual trend was observed in high sugar beverages. The geographical distribution of trends in diet-attributable HCKD burden varied globally from 1990 to 2021, with the highest burden observed in Southern Asia and Central Sub-Saharan Africa. Meanwhile, the diet-attributable HCKD burden increased with aging, reaching its peak in those aged 70. Decomposition analysis indicated that the diet-attributable HCKD burden is mainly caused by population growth, especially in low SDI regions. While the absolute number of mortality and DALYs has increased sharply, the relatively stable ASMR and ASDR suggest that this increase might be primarily driven by population growth. Compared to the absolute number, ASRs more effectively reflect changes in disease burden by eliminating the confounding effects of population size and age distribution.

Our analysis identified three dietary risk factors that contributed the most to diet-attributable CKD burden from 1990 to 2021: low fruit intake, low vegetable intake, and high sodium intake. The intake of fruits and vegetables remains below recommended levels in some countries despite substantial evidence supporting their health benefits. A considerable portion of Americans maintained a status of inadequate fruit and decreasing vegetable consumption since the publication of the Dietary Guidelines for Americans 2010 (16). Less than 20% of individuals meet the requirements of fruit and vegetable intake across Americans (17). Furthermore, the risks associated with low fruit and low vegetable intake are more prevalent in developing countries, mainly due to limited accessibility and affordability, which stands in contrast to the conventional view of their low cost. A research of approximately 0.2 million adult participants from 52 low- and middle-income countries revealed that 77% of respondents consumed less than the minimum recommendation of fruit and vegetable consumption (18). Consuming processed meat can raise interleukin 6 (IL-6) and decrease tumor necrosis factor-alpha (TNF-*α*), leading to an increase of oxidative stress and inflammation (19), which are implicated in the pathogenesis of CKD. High processed meat consumption is often accompanied by high sodium intake, which has detrimental effects on kidney function, including hyperfiltration with filtration fraction and glomerular pressure (20). Kidney Disease Improving Global Outcomes suggested a reduction to 2 g/day of sodium (equivalent to 5 g/day of salt) for adult CKD patients (21). Growing evidence has proved the role of moderate dietary sodium restriction in the management of kidney protection and hypertension. Hence, reduction of sodium is crucial in the control of the global HCKD burden.

SDI is a comprehensive indicator to represent the society and economic development related to health outcomes. It is apparent that people in high SDI regions are more likely to benefit from quality education, established healthcare, prioritized policies, and enhanced health assurances. In contrast, the absence of advanced medication hinders effective CKD screening and therapy among people in low SDI regions. In our study, we revealed that the regions with the biggest increasing trend of CKD burden were predominantly the High-income North America and the Southern Sub-Sahara Africa. In North America, mainly including the USA and Canada, hypertension is one of the major causes of CKD. The prevalence of hypertension is particularly severe in the USA, with 45.6% of the USA population affected by hypertension from the data of the National Health and Nutrition Examination Survey (22). Accumulating evidence has illustrated that a vegetarian diet may contribute to health improvement and prevention of chronic diseases (23), including hypertension and CKD (24). Consuming more plant-based food can reduce oxidative stress and chronic inflammation caused by chronic diseases. Our research indicated that the USA leads globally in the HCKD burden caused by low fruit, low vegetable, and high processed meat, with a rising trend over the years. This highlights that dietary patterns in the USA are associated with significant dietary risks, which may potentially contribute to increased HCKD burden.

Under the background of a globally increasing trend of diet-attributable HCKD burden (both ASMR and ASDR), the regions of High-income Asia Pacific and East Asia exhibited the opposite patterns in terms of HCKD burden trend in our study, mainly including Japan, Republic of Korea, Singapore and China. The traditional Asian diet is centered on health and balance, characterized by high contents of fibers, vitamins, fruits, vegetables and low contents of fat and meat (25). A randomized controlled trial study showed that an Asian dietary pattern improved insulin sensitivity and glucose metabolism, and induced weight loss and diabetes control (26). These characteristics are reflected in our findings, which revealed a slight reduction in HCKD burden attributable to low fruit, low vegetable, low whole grain and high sodium. Conversely, the HCKD burden attributable to high processed meat and high sugar beverages has not demonstrated the anticipated decline, but instead exhibited a gradual upward trend. This trend may be related to the rising consumption of wheat, animal protein, and sugars in Asian dietary patterns in recent years (27). Many adults in India prefer processed foods that are high in meat, sugar and sodium, which has coincided with an increase in non-communicable diseases related to dietary risk factors, including hypertension and hyperglycemia (28). Furthermore, we also observed a rapid increase trend of the HCKD burden caused by high sugar beverages and high sodium in India, which was significantly different from the situation in other Asian countries. The diet-attributable HCKD burden is anticipated to increase further in the absence of effective interventions to control HCKD (29).

Older adults are more vulnerable to hypertension and CKD, with aging itself recognized as a separate and critical risk factor for CKD progression based on the previous evidence (30, 31). As a natural consequence of aging, the physiological decline in renal function increasingly affects older adults, including a decrease in nephron number and glomerular filtration rate (32). In our analysis, the diet-attributable HCKD burden caused by high sugar beverage consumption increased sharply among older adults, particularly in those over 70 years. Glycolipid metabolism is significantly dysregulated in CKD patients because of reduced expression of genes involved in fatty acids and glucose metabolism (33). Previous studies have shown that excessive fructose intake might increase the expression of Na-H exchanger-3 (NHE3) and promote sodium reabsorption in the renal tubules, leading to internal salt retention and kidney damage (34). Evidence from a Chinese cohort of older populations indicated that low vegetable and high dietary sodium intake were associated with accelerated eGFR decline (35). This association may be partially explained by age-related declines in renal function, including diminished acid excretion capacity and reduced clearance of reactive oxygen species (ROS). The substantial CKD burden caused by high sugar beverages among older adults seems to be attributed to the metabolic disorder. Nevertheless, the prevalence of CKD significantly decreased in older adults with high dietary diversity and sufficient contents of vegetables, fruits, fish, and soy products (36). Regular consumption of dairy products has also been associated with improved renal function among older adults by reducing inflammation, oxidative stress, and endothelial dysfunction (37). Different sources of protein might affect renal function in older adults, with plant-based protein exerting a smaller impact than animal-based protein (38). These all suggest that older adults need a comprehensive and high-quality diet to delay the progression of CKD, reduce mortality risk, and enhance life quality.

Compared with previous studies that mainly focused on total CKD burden and total risk factors (39–42), we quantitatively assessed the contributions of various dietary risks to HCKD burden both individually and collectively. The refined integration of dietary risks and HCKD burden, along with systematic spatiotemporal trend analysis, may address certain gaps in existing research and deliver reliable assessments of HCKD burden. Admittedly, there were several limitations in this study. Firstly, the lack of reliable diagnostic capacity in some undeveloped and developing countries may result in wide uncertainty intervals of the estimation of HCKD burden. Secondly, inconsistent diagnostic criteria across countries might have affected the accuracy of HCKD classification. Lastly, most HCKD cases were derived from cross-sectional data, and reporting inaccuracies may have contributed to a potential overestimation of HCKD burden. Despite these limitations, this study provides a thoroughgoing perception of CKD burden using up-to-date data and superior methods from the past to the present.

In conclusion, diet-attributable HCKD remains an ongoing issue of global public health, especially in middle- and low-SDI regions and countries. The HCKD caused by low fruit and high sugar beverages had the biggest burden and the highest increasing trend, respectively. Older adults need to pay more attention to monitor dietary risks impacting kidney health than the youth. On a global scale, dietary risks remain largely overlooked and undervalued, leading to an increasing burden of related diseases, which gravely threaten public health. In the future, public health strategies should prioritize increasing the intake of fruits and vegetables through coordinated efforts that combine educational campaigns, targeted subsidies, and improvements in food distribution systems to enhance both accessibility and affordability. By contrast, immediate policy actions are warranted to curb excessive sugar-sweetened beverages and high-sodium foods intake. Effective interventions may include taxation and regulatory approaches that limit the accessibility of these products.

## Data Availability

Publicly available datasets were analyzed in this study. This data can be found at: https://vizhub.healthdata.org/gbd-results/.
