# Supplementary material for: Global burden of chronic kidney disease due to hypertension attributable to dietary risks in adults aged 25 years and older: findings from the global burden of disease study 2021
Source: Front Nutr. 2025 Jul 24;12:1593057. doi: 10.3389/fnut.2025.1593057 (PMC12328163; doi:10.3389/fnut.2025.1593057)
Supplement: Supplementary file 2 [file Data_Sheet_1.pdf]

Supplementary Materials:

Contents:

Supplementary Table 1. Percentage change of diet-attributable HCKD burden from 1990 to 2021
Supplementary Table 2. Number and age-standardized rates with rates changes attributable to dietary risks for HCKD burden across 5 SDI regions from 1990 to 2021
Supplementary Table 3. Number and age-standardized rates with rates changes attributable to dietary risks for HCKD burden across gender from 1990 to 2021
Supplementary Table 4. Number and age-standardized rates with rates changes attributable to dietary risks for HCKD burden across age group from 1990 to 2021
Supplementary Table 5. Changes in mortality and DALYs number of diet-attributable HCKD according to population-level determinants and causes from 1990 to 2021

Supplementary Figure 1. AAPC for diet-attributable HCKD from 1990 to 2021 across global and different SDI regions. (A). AAPC of ASMR. (B). AAPC of ASDR.

Supplementary Table 1. Percentage change of diet-attributable HCKD burden from 1990 to 2021

Dietary risks	Percentage change (%)					
	Global	High SDI	High-middle SDI	Middle SDI	Low-middle SDI	Low SDI
Mortality						
total dietary risk	176.0	275.1	121.3	176.7	168.4	118.8
low fruits	164.1	253.8	103.5	164.4	156.4	123.6
low vegetables	155.6	291.3	64.6	150.0	154.9	112.3
low whole grains	323.4	382.2	233.5	388.9	350.3	162.9
high processed meat	401.0	497.3	197.7	455.0	465.8	180.8
high sugar beverages	489.8	563.3	279.4	572.7	800.9	169.6
high sodium	171.9	245.7	140.3	171.7	181.0	92.9
DALYs						
total dietary risk	136.8	196.6	77.9	140.4	146.0	114.6
low fruits	127.6	177.7	58.6	130.4	137.6	120.1
low vegetables	119.9	207.0	23.0	117.0	133.0	108.8
low whole grains	295.4	343.4	187.6	350.9	374.2	179.1
high processed meat	335.4	435.6	115.0	420.3	523.3	197.9
high sugar beverages	441.4	524.1	233.4	481.5	734.9	164.3
high sodium	138.0	181.0	107.1	140.5	159.1	86.6

Supplementary Table 2. Number and age-standardized rates with rates changes attributable to dietary risks for HCKD burden across 5 SDI regions from 1990 to 2021

Regions	Mortality					DALYs				
	1990		2021		1990-2021	1990		2021		1990-2021
	Number No.×10^3 (95%UI)	ASMR Per 10^5 (95%UI)	Number No.×10^3 (95%UI)	ASMR Per 10^5 (95%UI)	AAPC (95%CI)	Number No.×10^3 (95%UI)	ASDR Per 10^5 (95%UI)	Number No.×10^3 (95%UI)	ASDR Per 10^5 (95%UI)	AAPC (95%CI)
High SDI										
total dietary risk	11.3 (6.5, 17.2)	1.9 (1.1, 2.9)	42.5 (23.4, 64.4)	3.2 (1.7, 4.8)	1.7 (1.4, 1.9)$^+$	259.6 (158.9, 378.6)	43.7 (26.6, 63.8)	770.1 (443.6, 1130.7)	67.0 (38.5, 98.5)	1.4 (1.2, 1.6)$^+$
low fruits	5.7 (3.2, 8.8)	1.0 (0.5, 1.5)	20.2 (10.6, 31.3)	1.5 (0.8, 2.4)	1.5 (1.2, 1.7)$^+$	135.8 (80.2, 203.3)	23.0 (13.5, 34.5)	377.0 (201.8, 572.5)	33.5 (17.7, 51.3)	1.2 (1.0, 1.4)$^+$
low vegetables	4.4 (2.2, 7.2)	0.7 (0.4, 1.2)	17.1 (8.6, 28.3)	1.3 (0.6, 2.1)	1.8 (1.5, 2.1)$^+$	98.3 (53.0, 159.4)	16.6 (8.9, 26.9)	301.8 (153.1, 494.3)	26.2 (13.1, 43.3)	1.5 (1.3, 1.7)$^+$
low whole grains	0.2 (0, 0.5)	0 (0, 0.1)	0.8 (0.1, 2)	0.1 (0, 0.1)	2.2 (2.0, 2.4)$^+$	2.7 (0.2, 8.9)	0.5 (0, 1.5)	12.1 (1.3, 32.5)	1.0 (0.1, 2.8)	2.5 (2.3, 2.7)$^+$

high processed meat	0.3 (0, 0.9)	0.1 (0, 0.2)	1.6 (0.2, 4.3)	0.1 (0, 0.3)	2.9 (2.6, 3.3)+	5.1 (0.2, 19.4)	0.9 (0, 3.3)	27.1 (2.0, 83.1)	2.5 (0.2, 7.7)	3.4 (3.1, 3.7)+
high sugar beverages	0.1 (0, 0.2)	0 (0, 0)	0.4 (0.1, 1)	0 (0, 0.1)	3.4 (3.2, 3.7)+	1.2 (0.1, 4.2)	0.2 (0, 0.7)	7.2 (0.7, 20.7)	0.7 (0.1, 2.1)	4.3 (4.0, 4.5)+
high sodium	2.9 (0.4, 8.6)	0.5 (0.1, 1.4)	10.0 (0.6, 31.9)	0.8 (0, 2.4)	1.5 (1.1, 1.8)+	67.0 (9.4, 185.3)	11.1 (1.6, 30.9)	188.2 (12.7, 576.9)	16.0 (1.1, 48.9)	1.2 (0.9, 1.5)+
High-middle SDI										
total dietary risk	10.1 (5.2, 16.8)	2.2 (1.1, 3.6)	22.4 (11.0, 38.6)	2.2 (1.1, 3.7)	0 (-0.2, 0.1)	288.5 (154.1, 462.6)	54.9 (29.5, 87.4)	513.2 (264.1, 857.5)	48.7 (25.1, 81.3)	-0.4 (-0.5, -0.3)+
low fruits	4.6 (2.2, 7.7)	1.0 (0.5, 1.6)	9.3 (4.6, 15.3)	0.9 (0.5, 1.5)	-0.3 (-0.4, -0.1)*	136.2 (70.7, 221.9)	25.8 (13.4, 41.8)	215.9 (112.3, 347.7)	20.8 (10.8, 33.6)	-0.7 (-0.8, -0.6)+
low vegetables	3.4 (1.6, 6.1)	0.8 (0.4, 1.3)	5.6 (2.7, 9.8)	0.6 (0.3, 1.0)	-1.0 (-1.2, -0.8)+	93.8 (45.1, 164.7)	18.1 (8.8, 31.6)	115.4 (58.7, 195.4)	11.2 (5.7, 19.1)	-1.6 (-1.7, -1.4)+
low whole grains	0.1 (0, 0.4)	0 (0, 0.1)	0.4 (0.1, 1.2)	0 (0, 0.1)	0.7 (0.5, 1.0)+	2.5 (0.2, 8.1)	0.6 (0, 1.7)	7.3 (0.7, 21.3)	0.7 (0.1, 2.1)	0.7 (0.5, 0.9)+
high processed meat	0.1 (0, 0.4)	0 (0, 0.1)	0.3 (0, 0.9)	0 (0, 0.1)	0.5 (0.2, 0.7)+	2.7 (0.1, 9.8)	0.6 (0, 2)	5.8 (0.5, 16.6)	0.6 (0.1, 1.6)	-0.1 (-0.3, 0.1)
high sugar beverages	0 (0, 0.1)	0 (0, 0)	0.1 (0, 0.3)	0 (0, 0)	1.2 (0.9, 1.4)+	0.7 (0, 2.3)	0.2 (0, 0.5)	2.4 (0.3, 6.7)	0.2 (0, 0.7)	1.4 (1.2, 1.6)+
high sodium	4.1 (0.8, 10.2)	0.8 (0.2, 2.1)	9.8 (1.6, 25.7)	0.9 (0.1, 2.4)	0.3 (0.2, 0.5)+	116.7 (24.9, 278.9)	21.7 (4.5, 52.4)	241.8 (47.0, 592.9)	22.3 (4.3, 55.1)	0.1 (0, 0.2)
Middle SDI										
total dietary risk	25.8 (13.1, 41.5)	5.7 (2.9, 9.1)	71.5 (36.6, 113.8)	5.4 (2.8, 8.6)	-0.2 (-0.2, -0.1)+	752.8 (387.4, 1188.3)	133.2 (69.3, 207.8)	1809.7 (958.0, 2844.1)	124.5 (66.0, 195.5)	-0.2 (-0.3, -0.1)+
low fruits	11.5 (5.3, 19.3)	2.5 (1.2, 4.2)	30.4 (14.9, 48.8)	2.3 (1.1, 3.7)	-0.3 (-0.3, -0.2)+	347.6 (165.4, 578.5)	60.4 (29.1, 99.0)	800.6 (400.3, 1279.0)	55.1 (27.6, 87.8)	-0.3 (-0.4, -0.2)+
low vegetables	11.6 (5.4, 19.7)	2.6 (1.2, 4.4)	29.1 (14.4, 47.8)	2.2 (1.1, 3.7)	-0.5 (-0.7, -0.4)+	336.9 (156.2, 573.9)	59.9 (28.1, 100.9)	731.4 (366.7, 1209.8)	50.6 (25.3, 83.5)	-0.5 (-0.7, -0.4)+
low whole grains	0.1 (0, 0.4)	0 (0, 0.1)	0.6 (0.1, 1.7)	0.1 (0, 0.1)	1.0 (0.9, 1.1)+	2.9 (0.1, 10.0)	0.6 (0, 2.1)	13.1 (1.0, 39.0)	1.0 (0.1, 2.8)	1.4 (1.2, 1.6)+

high processed meat	0 (0, 0.1)	0 (0, 0)	0.2 (0, 0.6)	0 (0, 0.1)	1.6 (1.3, 1.8)$^{+}$	1.0 (0, 3.4)	0.2 (0, 0.6)	5.2 (0.3, 15.3)	0.4 (0, 1.1)	2.1 (1.7, 2.4)$^{+}$
high sugar beverages	0 (0, 0.1)	0 (0, 0)	0.2 (0, 0.6)	0 (0, 0)	2.5 (2.1, 2.9)$^{+}$	1.0 (0.1, 2.9)	0.2 (0, 0.5)	5.6 (0.5, 15.5)	0.4 (0, 1.1)	2.8 (2.4, 3.2)$^{+}$
high sodium	9.1 (1.6, 22.6)	1.9 (0.3, 4.9)	24.8 (3.3, 66.3)	1.8 (0.2, 5.0)	-0.2 (-0.3, -0.1)*	256.3 (48.7, 619.6)	45.8 (8.3, 112.0)	616.4 (92.3, 1593.5)	41.8 (6.0, 109.2)	-0.3 (-0.4, -0.2)$^{+}$
Low-middle SDI										
total dietary risk	14.5 (7.6, 22.9)	5.1 (2.7, 8.0)	39.0 (21.5, 60.9)	5.7 (3.1, 8.8)	0.3 (0.2, 0.5)$^{+}$	455.6 (244.5, 701.5)	130.8 (71.4, 199.1)	1120.9 (630.3, 1717.8)	139.1 (79.0, 211.6)	0.2 (0.2, 0.3)$^{+}$
low fruits	8.1 (3.8, 13.5)	2.8 (1.3, 4.6)	20.7 (10.6, 33.2)	3.0 (1.5, 4.7)	0.3 (0.1, 0.5)*	262.8 (126.8, 432.2)	73.9 (36.4, 119.9)	624.5 (321.9, 989.5)	76.2 (39.9, 119.7)	0.1 (0.1, 0.2)$^{+}$
low vegetables	7.4 (3.5, 12.5)	2.6 (1.2, 4.4)	18.8 (9.5, 31.1)	2.7 (1.4, 4.5)	0.2 (0, 0.3)*	233.6 (110.6, 397.6)	66.8 (32.2, 112.0)	544.3 (276.2, 897.3)	67.4 (34.6, 110.2)	0 (0, 0.1)
low whole grains	0.1 (0, 0.3)	0 (0, 0.1)	0.4 (0, 1.0)	0.1 (0, 0.2)	1.4 (1.2, 1.6)$^{+}$	1.8 (0.1, 6.5)	0.7 (0, 2.2)	8.6 (0.6, 26.0)	1.2 (0.1, 3.4)	1.8 (1.7, 1.9)$^{+}$
high processed meat	0 (0, 0.1)	0 (0, 0)	0.1 (0, 0.4)	0 (0, 0.1)	2.1 (1.9, 2.3)$^{+}$	0.6 (0, 2.4)	0.2 (0, 0.8)	3.4 (0.1, 12.0)	0.4 (0, 1.5)	2.7 (2.5, 2.9)$^{+}$
high sugar beverages	0 (0, 0)	0 (0, 0)	0.1 (0, 0.2)	0 (0, 0)	3.9 (3.5, 4.3)$^{+}$	0.2 (0, 0.7)	0.1 (0, 0.2)	1.5 (0.1, 4.8)	0.2 (0, 0.6)	4.1 (3.8, 4.4)$^{+}$
high sodium	3.2 (0.3, 9.2)	1.1 (0.1, 3.3)	8.9 (0.6, 27.4)	1.3 (0.1, 4.0)	0.5 (0.3, 0.7)$^{+}$	93.2 (10.4, 267.7)	27.7 (3.0, 79.8)	241.5 (18.1, 725.8)	30.6 (2.2, 92.1)	0.4 (0.2, 0.5)$^{+}$
Low SDI										
total dietary risk	8.4 (4.3, 13.3)	8.5 (4.4, 13.3)	18.3 (9.8, 28.7)	8.4 (4.5, 13.0)	0 (-0.1, 0.1)	237.7 (123.7, 373.5)	191.5 (100.7, 297.7)	510.0 (277.3, 794.7)	180.6 (98.9, 277.4)	-0.2 (-0.3, -0.1)$^{+}$
low fruits	4.4 (2.0, 7.5)	4.4 (2.0, 7.3)	9.9 (4.7, 16.2)	4.4 (2.1, 7.1)	0.1 (-0.1, 0.2)	130.1 (60.5, 219.4)	102.3 (48.1, 170.2)	286.4 (138.7, 468.2)	98.7 (48.6, 158.6)	-0.1 (-0.2, 0)*
low vegetables	4.9 (2.3, 8.4)	4.9 (2.3, 8.3)	10.3 (4.9, 17.4)	4.7 (2.2, 7.7)	-0.1 (-0.2, 0)	140.2 (65.5, 243.0)	111.4 (52.5, 190.8)	292.7 (142.3, 494.8)	101.9 (50.1, 170.1)	-0.3 (-0.4, -0.2)$^{+}$
low whole grains	0.1 (0, 0.2)	0.1 (0, 0.2)	0.2 (0, 0.4)	0.1 (0, 0.2)	0.2 (0, 0.4)	1.4 (0.1, 4.3)	1.3 (0.1, 3.8)	3.8 (0.2, 11.2)	1.4 (0.1, 4.1)	0.3 (0.2, 0.5)$^{+}$

high processed meat	0 (0, 0.1)	0 (0, 0.1)	0.1 (0, 0.2)	0 (0, 0.1)$^{+}$	0.4 (0.3, 0.5)	0.7 (0, 2.6)	0.7 (0, 2.1)	2.2 (0.1, 7.1)	0.8 (0.1, 2.4)	0.6 (0.5, 0.7)$^{+}$
high sugar beverages	0 (0, 0)	0 (0, 0)	0 (0, 0)	0 (0, 0)$^{+}$	0.6 (0.5, 0.8)	0.1 (0, 0.4)	0.1 (0, 0.3)	0.3 (0, 1.1)	0.1 (0, 0.3)	0.4 (0.3, 0.6)$^{+}$
high sodium	1.4 (0.1, 4.7)	1.5 (0.1, 4.9)	2.7 (0.1, 9.7)	1.3 (0, 4.6)$^{+}$	-0.3 (-0.5, -0.1)	35.7 (1.5, 119.3)	30.9 (1.3, 102.4)	66.6 (1.3, 237.1)	26.2 (0.6, 91.7)	-0.5 (-0.7, -0.4)$^{+}$

Note: $^{+}$ means *P*-value < 0.001, Note: * means *P*-value < 0.05.

Supplementary Table 3. Number and age-standardized rates with rates changes attributable to dietary risks for HCKD burden across gender from 1990 to 2021

Risks	Mortality					DALYs				
	1990		2021		1990-2021	1990		2021		1990-2021
	Number No.×10^3 (95%UI)	ASMR Per 10^5 (95%UI)	Number No.×10^3 (95%UI)	ASMR Per 10^5 (95%UI)	AAPC (95%CI)	Number No.×10^3 (95%UI)	ASDR Per 10^5 (95%UI)	Number No.×10^3 (95%UI)	ASDR Per 10^5 (95%UI)	AAPC (95%CI)
Both										
total dietary risk	70.3 (37.2, 110.9)	3.7 (2.0, 5.7)	193.9 (104.3, 301.3)	4.3 (2.3, 6.7)	0.6 (0.4, 0.7)$^{+}$	1996.3 (1081.1, 3070.4)	91.7 (50.1, 140.1)	4728.1 (2616.8, 7223.5)	101.2 (56.0, 154.5)	0.3 (0.2, 0.4)$^{+}$
low fruits	34.3 (16.7, 56.3)	1.8 (0.9, 2.9)	90.5 (46.1, 142.2)	2.0 (1.0, 3.2)	0.4 (0.3, 0.5)$^{+}$	1013.3 (508.2, 1650.3)	46.0 (23.3, 74.3)	2306.1 (1198.7, 3603.8)	49.4 (25.7, 77.3)	0.2 (0.2, 0.3)$^{+}$
low vegetables	31.7 (15.2, 53.6)	1.7 (0.8, 2.8)	81.0 (40.6, 132.4)	1.8 (0.9, 2.9)	0.3 (0.2, 0.4)$^{+}$	903.8 (436.9, 1525.6)	41.3 (20.1, 69.3)	1987.6 (1004.4, 3265.2)	42.6 (21.5, 70.1)	0.1 (0, 0.2)*
low whole grains	0.5 (0, 1.7)	0 (0, 0.1)	2.3 (0.3, 6.2)	0.1 (0, 0.1)	0.5 (0.4, 0.7)$^{+}$	11.4 (0.7, 37.9)	0.6 (0, 2)	44.9 (4.0, 128.9)	1.0 (0.1, 2.8)	0.4 (0.2, 0.5)$^{+}$
high processed meat	0.5 (0, 1.6)	0 (0, 0.1)	2.3 (0.2, 6.3)	0.1 (0, 0.1)	1.1 (0.9, 1.2)$^{+}$	10.0 (0.5, 37.3)	0.6 (0, 1.9)	43.7 (3.3, 132.2)	1.0 (0.1, 2.9)	0.6 (0.4, 0.7)$^{+}$
high sugar beverages	0.1 (0, 0.4)	0 (0, 0)	0.8 (0.1, 2)	0 (0, 0)	2.0 (1.8, 2.2)$^{+}$	3.1 (0.2, 10.3)	0.2 (0, 0.5)	17.0 (1.8, 47.8)	0.4 (0, 1.0)	1.9 (1.8, 2.1)$^{+}$
high sodium	20.7 (3.2, 54.6)	1.1 (0.2, 2.8)	56.3 (6.3, 159.6)	1.2 (0.1, 3.5)	0.5 (0.4, 0.6)$^{+}$	569.5 (97.7, 1454.2)	26.3 (4.4, 67.6)	1355.7 (178.6, 3672.6)	28.6 (3.7, 78.0)	0.2 (0.1, 0.3)$^{+}$
Male										
total dietary	38.8	0	105.0	0	0.4	1134.3	0.1	2683.0	0.1	0.3

risk	(20.2, 62.2)	(0, 0)	(55.4, 164.8)	(0, 0)	(0.3, 0.6)$^{+}$	(606.4, 1774.7)	(0.1, 0.2)	(1468.0, 4134.3)	(0.1, 0.2)	(0.2, 0.4)$^{+}$
low fruits	18.5 (8.7, 31.0)	0 (0, 0)	47.5 (23.8, 76.1)	0 (0, 0)	0.2 (0.1, 0.4)$^{+}$	565.5 (275.5, 932.4)	0.1 (0, 0.1)	1274.0 (648.2, 2009.0)	0.1 (0, 0.1)	0.1 (0.1, 0.2)$^{+}$
low vegetables	17.5 (8.1, 29.8)	0 (0, 0)	42.8 (20.9, 70.8)	0 (0, 0)	0.1 (0, 0.2)*	512.7 (242.5, 872.5)	0.1 (0, 0.1)	1108.3 (551.5, 1823.7)	0.1 (0, 0.1)	0 (-0.1, 0.2)
low whole grains	0.3 (0, 0.9)	0 (0, 0)	1.2 (0.1, 3.1)	0 (0, 0)	0.3 (0.2, 0.4)$^{+}$	6.4 (0.3, 21.0)	0 (0, 0)	23.4 (1.9, 67.9)	0 (0, 0)	0.2 (0.1, 0.3)*
high processed meat	0.2 (0, 0.8)	0 (0, 0)	1.2 (0.1, 3.3)	0 (0, 0)	1.0 (0.8, 1.2)$^{+}$	5.5 (0.3, 20.1)	0 (0, 0)	23.7 (1.6, 72.5)	0 (0, 0)	0.7 (0.6, 0.8)$^{+}$
high sugar beverages	0.1 (0, 0.2)	0 (0, 0)	0.4 (0, 1.0)	0 (0, 0)	1.9 (1.7, 2.1)$^{+}$	1.7 (0.1, 5.6)	0 (0, 0)	8.7 (0.8, 25.1)	0 (0, 0)	1.9 (1.7, 2.1)$^{+}$
high sodium	12.2 (2.1, 31.9)	0 (0, 0)	34.1 (4.5, 92.5)	0 (0, 0)	0.5 (0.3, 0.6)$^{+}$	343.0 (64.3, 866.6)	0 (0, 0.1)	847.8 (125.0, 2210.0)	0 (0, 0.1)	0.3 (0.2, 0.4)$^{+}$
Female										
total dietary risk	31.5 (16.8, 49.6)	0 (0, 0)	89.0 (47.7, 138.6)	0 (0, 0)	0.6 (0.5, 0.7)$^{+}$	862.0 (471.7, 1328.8)	0.1 (0, 0.1)	2045.2 (1132.4, 3141.8)	0.1 (0, 0.1)	0.3 (0.3, 0.4)$^{+}$
low fruits	15.7 (7.8, 25.9)	0 (0, 0)	43.0 (22.0, 67.4)	0 (0, 0)	0.5 (0.4, 0.5)$^{+}$	447.8 (229.8, 723.5)	0 (0, 0.1)	1032.1 (540.7, 1613.8)	0 (0, 0.1)	0.2 (0.1, 0.3)$^{+}$
low vegetables	14.2 (7.0, 24.2)	0 (0, 0)	38.1 (19.3, 62.5)	0 (0, 0)	0.5 (0.4, 0.6)$^{+}$	391.1 (192.8, 663.1)	0 (0, 0.1)	879.3 (451.5, 1442.6)	0 (0, 0.1)	0.2 (0.1, 0.3)$^{+}$
low whole grains	0.2 (0, 0.8)	0 (0, 0)	1.1 (0.1, 3.1)	0 (0, 0)	0.6 (0.5, 0.8)$^{+}$	5.0 (0.3, 16.7)	0 (0, 0)	21.5 (2.0, 61.2)	0 (0, 0)	0.4 (0.4, 0.5)$^{+}$
high processed meat	0.2 (0, 0.8)	0 (0, 0)	1.1 (0.1, 3.2)	0 (0, 0)	1.0 (0.8, 1.1)	4.6 (0.2, 17.4)	0 (0, 0)	20.0 (1.5, 61.2)	0 (0, 0)	0.4 (0.3, 0.6)$^{+}$
high sugar beverages	0.1 (0, 0.2)	0 (0, 0)	0.4 (0.1, 1.0)	0 (0, 0)	2.0 (1.8, 2.2)$^{+}$	1.4 (0.1, 4.9)	0 (0, 0)	8.3 (0.9, 23.0)	0 (0, 0)	2.0 (1.8, 2.1)$^{+}$
high sodium	8.5 (1.0, 23.4)	0 (0, 0)	22.2 (1.6, 68.5)	0 (0, 0)	0.3 (0.2, 0.4)$^{+}$	226.5 (31.2, 607.4)	0 (0, 0.1)	507.9 (45.5, 1493.0)	0 (0, 0.1)	0 (0, 0.1)

Note: $^{+}$ means *P*-value < 0.001, Note: * means *P*-value < 0.05.

Supplementary Table 4. Number and age-standardized rates with rates changes attributable to dietary risks for HCKD burden across age groups from 1990 to 2021

Age (years)	Mortality				DALYs			
	1990		2021		1990		2021	
	Number No.×10^3 (95%UI)	ASMR Per 10^5 (95%UI)	Number No.×10^3 (95%UI)	ASMR Per 10^5 (95%UI)	Number No.×10^3 (95%UI)	ASDR Per 10^5 (95%UI)	Number No.×10^3 (95%UI)	ASDR Per 10^5 (95%UI)
Both								
25-29	0.8 (0.4, 1.4)	0.2 (0.1, 0.3)	1.3 (0.6, 2.1)	0.2 (0.1, 0.4)	65.6 (35.0, 106.9)	14.8 (7.9, 24.1)	101.7 (57.0, 163.6)	17.3 (9.7, 27.8)
30-34	1.0 (0.5, 1.7)	0.3 (0.1, 0.4)	1.8 (0.9, 2.9)	0.3 (0.1, 0.5)	76.4 (38.4, 120.0)	19.8 (10.0, 31.1)	134.8 (74.5, 207.2)	22.3 (12.3, 34.3)
35-39	1.5 (0.7, 2.5)	0.4 (0.2, 0.7)	2.7 (1.3, 4.4)	0.5 (0.2, 0.8)	98.7 (49.6, 156.0)	28.0 (14.1, 44.3)	178.5 (91.1, 282.1)	31.8 (16.3, 50.3)
40-44	2.0 (0.9, 3.5)	0.7 (0.3, 1.2)	4.0 (1.8, 6.8)	0.8 (0.4, 1.4)	118.7 (57.0, 199.8)	41.4 (19.9, 69.7)	234.8 (116.0, 384.6)	46.9 (23.2, 76.9)
45-49	2.6 (1.2, 4.4)	1.1 (0.5, 1.9)	5.6 (2.7, 9.3)	1.2 (0.6, 2.0)	134.7 (67.3, 217.1)	58.0 (29.0, 93.5)	288.7 (150.0, 463.1)	61.0 (31.7, 97.8)
50-54	4.0 (2.0, 6.6)	1.9 (0.9, 3.1)	8.5 (4.2, 13.7)	1.9 (0.9, 3.1)	178.7 (91.8, 286.2)	84.1 (43.2, 134.6)	382.1 (205.0, 590.0)	85.9 (46.1, 132.6)
55-59	5.7 (2.8, 9.0)	3.1 (1.5, 4.9)	12.7 (6.5, 19.9)	3.2 (1.6, 5.0)	221.0 (114.3, 334.2)	119.3 (61.7, 180.5)	491.8 (263.4, 745.7)	124.3 (66.6, 188.4)
60-64	7.1 (3.8, 11.1)	4.4 (2.4, 6.9)	15.6 (9.0, 24.0)	4.9 (2.8, 7.5)	237.6 (132.8, 356.6)	147.9 (82.7, 222.0)	516.4 (299.6, 770.3)	161.3 (93.6, 240.7)
65-69	8.2 (4.6, 13.2)	6.6 (3.7, 10.7)	20.0 (11.3, 31.9)	7.3 (4.1, 11.6)	232.2 (134.7, 360.5)	187.9 (109.0, 291.6)	557.6 (318.4, 859.7)	202.1 (115.4, 311.7)
70-74	9.0 (4.6, 13.8)	10.6 (5.4, 16.3)	22.7 (12.2, 35.8)	11.1 (5.9, 17.4)	207.7 (112.5, 312.1)	245.4 (132.9, 368.6)	520.3 (294.9, 803.1)	252.7 (143.3, 390.2)
75-79	9.5 (5.2, 14.5)	15.4 (8.4, 23.6)	23.8 (13.0, 36.1)	18.0 (9.9, 27.4)	178.1 (101.9, 260.7)	289.3 (165.6, 423.5)	435.8 (244.7, 649.8)	330.5 (185.5, 492.7)
80-84	8.5 (4.8, 13.1)	24.1 (13.7, 37.2)	25.3 (14.2, 37.8)	28.9 (16.2, 43.1)	128.2 (76.4, 186.2)	362.3 (216.1, 526.2)	365.0 (206.4, 530.4)	416.8 (235.7, 605.6)
85-89	6.6 (3.7, 10.1)	43.5 (24.4, 66.6)	25.1 (13.7, 37.8)	54.9 (30.0, 82.6)	78.1 (46.0, 115.8)	516.6 (304.2, 766.5)	283.0 (164.5, 415.0)	619.1 (359.7, 907.7)
90-94	3.0 (1.6, 4.3)	69.4 (37.9, 101.5)	16.9 (8.9, 26.2)	94.3 (49.9, 146.6)	31.3 (18.0, 44.8)	730.6 (419.6, 1045.3)	165.7 (92.9, 249.9)	926.2 (519.5, 1397.1)
95+	1.0 (0.5, 1.5)	94.9 (50.1, 143.4)	8.0 (4.0, 12.6)	147.4 (74.0, 230.8)	9.4 (5.3, 13.7)	921.7 (520.1, 1341.0)	72.0 (38.3, 108.9)	1320.8 (702.8, 1998.7)
Male								
25-29	0.5 (0.2, 0.9)	0.2 (0.1, 0.4)	0.8 (0.4, 1.4)	0.3 (0.1, 0.5)	39.5 (21.4, 63.8)	17.8 (9.6, 28.7)	65.1 (36.0, 105.3)	21.9 (12.1, 35.4)
30-34	0.6 (0.3, 1.0)	0.3 (0.1, 0.5)	1.1 (0.5, 1.8)	0.4 (0.2, 0.6)	45.0 (22.3, 71.6)	23.1 (11.4, 36.7)	82.2 (45.7, 124.4)	26.9 (15.0, 40.7)
35-39	0.9	0.5	1.7	0.6	59.4	33.3	110.7	39.1

	(0.4, 1.5)	(0.2, 0.9)	(0.8, 2.8)	(0.3, 1.0)	(29.9, 95.5)	(16.7, 53.4)	(56.3, 173.3)	(19.9, 61.2)
40-44	1.2	0.8	2.5	1.0	71.2	48.6	143.1	56.7
	(0.5, 2.1)	(0.4, 1.5)	(1.1, 4.2)	(0.4, 1.7)	(33.6, 118.5)	(23.0, 81.0)	(67.6, 230.4)	(26.8, 91.4)
45-49	1.5	1.3	3.3	1.4	79.0	66.7	168.6	70.9
	(0.7, 2.6)	(0.6, 2.2)	(1.6, 5.6)	(0.7, 2.4)	(39.3, 129.1)	(33.2, 109.0)	(85.9, 272.2)	(36.1, 114.4)
50-54	2.3	2.2	5.0	2.3	104.8	97.3	226.2	101.9
	(1.2, 3.9)	(1.1, 3.6)	(2.5, 8.3)	(1.1, 3.7)	(54.0, 167.7)	(50.2, 155.8)	(123.9, 354.7)	(55.8, 159.8)
55-59	3.3	3.6	7.4	3.8	128.4	138.2	286.3	147.0
	(1.6, 5.2)	(1.7, 5.6)	(3.7, 11.6)	(1.9, 6.0)	(66.2, 200.7)	(71.3, 216.1)	(149.2, 440.0)	(76.6, 226.0)
60-64	4.2	5.3	9.2	5.9	140.0	178.2	304.8	196.0
	(2.2, 6.7)	(2.8, 8.5)	(5.1, 14.4)	(3.3, 9.2)	(78.2, 212.9)	(99.6, 271.0)	(177.8, 458.6)	(114.3, 294.9)
65-69	4.7	8.2	11.6	8.8	133.3	232.5	323.6	245.5
	(2.6, 7.7)	(4.6, 13.5)	(6.3, 18.5)	(4.8, 14.0)	(76.9, 211.5)	(134.1, 369.0)	(185.5, 503.4)	(140.7, 381.9)
70-74	5.2	13.8	13.2	13.7	118.8	315.8	301.5	312.8
	(2.7, 8.2)	(7.1, 21.8)	(6.9, 20.9)	(7.2, 21.7)	(63.6, 181.7)	(169.0, 483.0)	(167.2, 470.5)	(173.5, 488.1)
75-79	5.2	20.6	13.3	22.2	96.6	382.8	243.3	407.0
	(2.8, 8.3)	(11.0, 32.8)	(7.2, 20.5)	(12.1, 34.3)	(52.7, 146.2)	(208.7, 579.4)	(135.1, 364.1)	(226.0, 609.0)
80-84	4.4	33.1	13.3	36.2	65.4	492.0	191.2	521.5
	(2.5, 6.8)	(18.6, 51.2)	(7.2, 20.0)	(19.7, 54.5)	(38.2, 97.1)	(287.4, 731.1)	(106.8, 280.4)	(291.3, 764.9)
85-89	3.1	60.8	12.4	71.7	36.3	716.5	139.1	806.4
	(1.7, 4.8)	(32.9, 93.8)	(6.7, 19.2)	(38.9, 111.3)	(20.7, 53.7)	(409.5, 1059.7)	(77.8, 209.2)	(451.2, 1212.5)
90-94	1.3	101.8	7.4	126.7	13.3	1059.7	72.5	1243.8
	(0.7, 1.9)	(54.7, 154.7)	(3.9, 11.5)	(66.6, 196.5)	(7.5, 19.6)	(598.6, 1556.8)	(39.6, 110.6)	(680.0, 1898.0)
95+	0.3	133.2	2.7	178.9	3.4	1297.6	24.6	1628.4
	(0.2, 0.5)	(69.1, 207.3)	(1.4, 4.2)	(93.0, 280.0)	(1.8, 5.0)	(697.6, 1905.1)	(13.5, 37.2)	(891.7, 2459.7)
Female								
25-29	0.3	0.1	0.4	0.1	26.1	11.8	36.6	12.6
	(0.1, 0.6)	(0.1, 0.3)	(0.2, 0.8)	(0.1, 0.3)	(13.4, 43.7)	(6.1, 19.9)	(20.2, 59.9)	(6.9, 20.6)
30-34	0.4	0.2	0.7	0.2	31.4	16.5	52.6	17.6
	(0.2, 0.7)	(0.1, 0.4)	(0.3, 1.1)	(0.1, 0.4)	(15.7, 51.4)	(8.3, 27.0)	(27.8, 82.8)	(9.3, 27.7)
35-39	0.6	0.3	1.0	0.3	39.2	22.6	67.8	24.4
	(0.3, 1.0)	(0.1, 0.6)	(0.5, 1.6)	(0.2, 0.6)	(19.2, 62.9)	(11.1, 36.3)	(34.5, 107.3)	(12.4, 38.6)
40-44	0.8	0.6	1.5	0.6	47.6	33.9	91.7	37.0
	(0.4, 1.4)	(0.3, 1.0)	(0.7, 2.6)	(0.3, 1.1)	(23.8, 80.4)	(16.9, 57.3)	(45.6, 148.6)	(18.4, 59.9)
45-49	1.1	0.9	2.3	1.0	55.7	48.9	120.1	51.0
	(0.5, 1.8)	(0.5, 1.6)	(1.1, 3.9)	(0.5, 1.6)	(27.9, 90.2)	(24.5, 79.2)	(60.3, 192.8)	(25.6, 81.8)
50-54	1.6	1.6	3.5	1.6	74.0	70.5	155.9	69.9
	(0.8, 2.7)	(0.8, 2.6)	(1.7, 5.7)	(0.8, 2.5)	(38.0, 116.2)	(36.2, 110.8)	(84.4, 246.0)	(37.9, 110.3)
55-59	2.4	2.6	5.3	2.7	92.6	100.3	205.4	102.2
	(1.2, 3.8)	(1.3, 4.2)	(2.8, 8.4)	(1.4, 4.2)	(47.1, 142.7)	(51.0, 154.6)	(112.1, 314.0)	(55.7, 156.2)
60-64	2.9	3.5	6.4	3.9	97.6	119.0	211.5	128.6
	(1.6, 4.6)	(2.0, 5.6)	(3.7, 9.8)	(2.3, 6.0)	(55.8, 148.3)	(68.0, 180.7)	(122.9, 314.9)	(74.7, 191.4)
65-69	3.5	5.2	8.4	5.8	98.9	149.3	234.0	162.5
	(1.9, 5.7)	(2.8, 8.6)	(4.6, 13.5)	(3.2, 9.4)	(57.2, 154.4)	(86.3, 233.0)	(132.0, 369.4)	(91.7, 256.5)

70-74	3.8 (1.9, 6.1)	8.0 (4.1, 12.9)	9.5 (5.2, 15.2)	8.7 (4.7, 13.8)	88.9 (48.8, 136.1)	189.0 (103.7, 289.3)	218.7 (126.8, 339.3)	199.8 (115.9, 310.0)
75-79	4.3 (2.4, 6.4)	11.8 (6.5, 17.7)	10.5 (5.7, 16.1)	14.5 (7.9, 22.4)	81.5 (47.8, 117.6)	224.3 (131.6, 323.7)	192.5 (108.3, 289.5)	267.0 (150.3, 401.5)
80-84	4.1 (2.3, 6.2)	18.7 (10.6, 27.9)	12.0 (6.8, 18.1)	23.6 (13.4, 35.5)	62.8 (38.1, 91.1)	284.3 (172.5, 412.3)	173.9 (99.5, 253.0)	341.4 (195.4, 496.7)
85-89	3.5 (2.0, 5.3)	34.8 (19.7, 52.4)	12.7 (6.9, 19.0)	44.7 (24.2, 66.8)	41.8 (25.1, 59.9)	415.8 (250.0, 596.1)	143.9 (81.2, 210.7)	505.5 (285.2, 740.0)
90-94	1.7 (0.9, 2.5)	55.9 (30.7, 82.3)	9.5 (4.9, 14.4)	78.6 (40.3, 119.4)	18.0 (10.4, 25.2)	593.8 (342.8, 833.5)	93.2 (52.5, 140.7)	772.7 (435.1, 1166.7)
95+	0.6 (0.3, 0.9)	81.7 (40.9, 125.1)	5.3 (2.6, 8.5)	135.3 (66.8, 214.9)	6.0 (3.3, 8.8)	792.6 (438.9, 1161.7)	47.4 (24.2, 73.1)	1202.7 (614.9, 1855.2)

Supplementary Table 5. Changes in mortality and DALYs number of diet-attributable HCKD according to population-level determinants and causes from 1990 to 2021

Location	Gender	Overall difference	Change due to population-level determinants (% contribute to the overall change)		
			Aging	Epidemiological change	Population
Mortality					
Global	Both	983418.8	17422.7 (1.8%)	293521.6 (29.8%)	672474.5 (68.4%)
	Male	566966.6	5641.6 (1.0%)	114081.5 (20.1%)	447243.5 (78.9%)
	Female	440602.5	13529.0 (3.1%)	145462.1 (33.0%)	281611.4 (63.9%)
High SDI	Both	148272.2	-45425.6 (-30.6%)	131111.7 (88.4%)	62586.2 (42.2%)
	Male	89352.2	-32104.9 (-35.9%)	76191.9 (85.3%)	45265.2 (50.7%)
	Female	62546.1	-19014.5 (-30.4%)	56444.5 (90.2%)	25116.0 (40.2%)
High-middle SDI	Both	32091.9	10257.8 (32.0%)	1957.6 (6.1%)	19876.5 (61.9%)
	Male	19369.5	8589.1 (44.3%)	-2161.8 (-11.2%)	12942.2 (66.8%)
	Female	13833.4	3479.0 (25.1%)	2018.1 (14.6%)	8336.3 (60.3%)
Middle SDI	Both	71057.8	28156.1 (39.6%)	-4190.0 (-5.9%)	47091.6 (66.3%)
	Male	39424.0	15488.9 (39.3%)	-1015.5 (-2.6%)	24950.6 (63.3%)
	Female	32306.5	13038.5 (40.4%)	-3058.1 (-9.5%)	22326.1 (69.1%)
Low-middle SDI	Both	37858.9	6217.6 (16.4%)	3848.0 (10.2%)	27793.3 (73.4%)
	Male	20765.1	2751.0 (13.2%)	2543.8 (12.3%)	15470.3 (74.5%)
	Female	17289.8	3265.6 (18.9%)	1770.3 (10.2%)	12254.0 (70.9%)
Low SDI	Both	175022.1	5363.8 (3.1%)	11043.7 (6.3%)	158614.7 (90.6%)
	Male	96573.0	3694.7 (3.8%)	1601.2 (1.7%)	91277.1 (94.5%)
	Female	80015.4	1996.6 (2.5%)	8500.0 (10.6%)	69518.8 (86.9%)
DALYs					
Global	Both	2731844.0	583893.5 (21.4%)	299007.6 (10.9%)	1848942.9 (67.7%)
	Male	1548647.9	362166.7 (23.4%)	144031.2 (9.3%)	1042450.1 (67.3%)
	Female	1183196.1	243090.0 (20.5%)	135001.3 (11.4%)	805104.8 (68.0%)
High SDI	Both	1011189.3	246915.0 (24.4%)	317766.6 (31.4%)	446507.7 (44.2%)
	Male	593920.1	198788.3 (33.5%)	104799.7 (17.6%)	290332.1 (48.9%)

	Female	427901.8	86482.8 (20.2%)	154631.9 (36.1%)	186787.1 (43.7%)
	Both	451501.7	138613.9 (30.7%)	-48104.0 (-10.7%)	360991.8 (80.0%)
High-middle SDI	Male	272039.9	110082.6 (40.5%)	-67999.8 (-25.0%)	229957.1 (84.5%)
	Female	189639.9	49270.9 (26.0%)	-9136.2 (-4.8%)	149505.2 (78.8%)
	Both	1400118.0	436642.4 (31.2%)	-114063.1 (-8.1%)	1077538.7 (77.0%)
Middle SDI	Male	804915.7	243372.7 (30.2%)	-26039.2 (-3.2%)	587582.3 (73.0%)
	Female	603499.5	198165.8 (32.8%)	-84915.3 (-14.1%)	490249.0 (81.2%)
	Both	877879.9	97142.6 (11.1%)	61647.0 (7.0%)	719090.3 (81.9%)
Low-middle SDI	Male	495772.0	43667.9 (8.8%)	46002.5 (9.3%)	406101.6 (81.9%)
	Female	385367.4	50140.9 (13.0%)	25346.7 (6.6%)	309879.7 (80.4%)
	Both	188473.4	-12811.0 (-6.8%)	-170412.6 (-90.4%)	371697.0 (197.2%)
Low SDI	Male	100852.4	-12854.0 (-12.7%)	-104088.3 (-103.2%)	217794.6 (216.0%)
	Female	87118.2	-1486.8 (-1.7%)	-64163.8 (-73.7%)	152768.8 (175.4%)

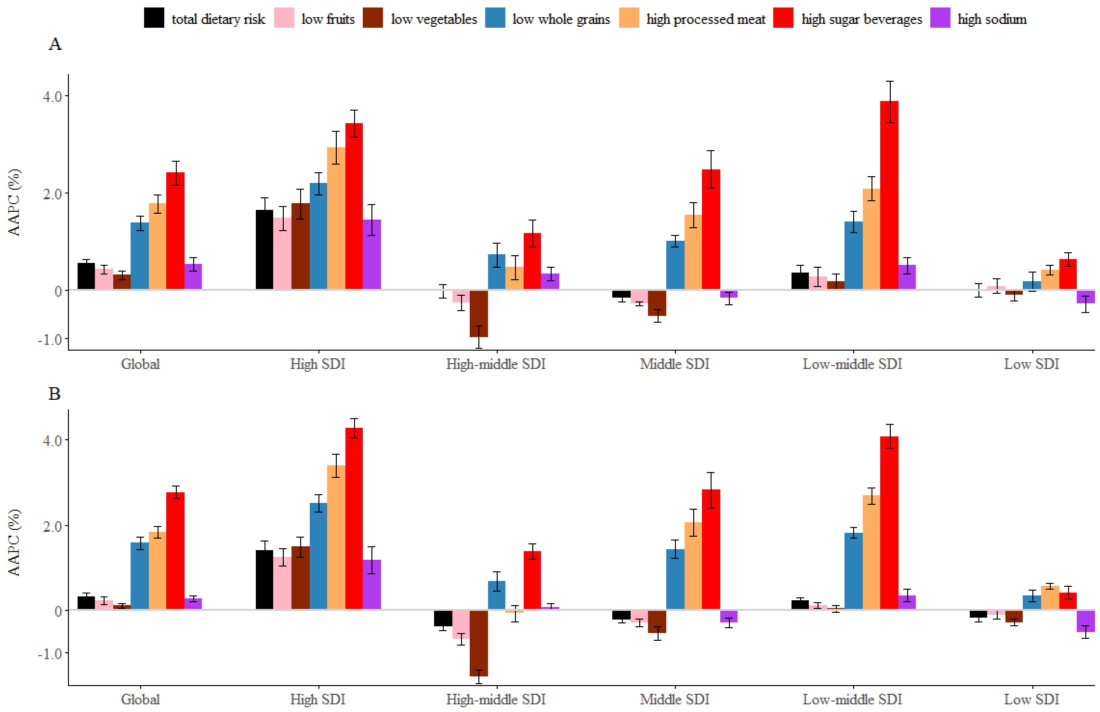

Supplementary Figure 1. AAPC for diet-attributable HCKD from 1990 to 2021 across global and different SDI regions. (A). AAPC of ASMR. (B). AAPC of ASDR.
